# Analysis of chromatin accessibility in human epidermis identifies putative barrier dysfunction-sensing enhancers

**DOI:** 10.1371/journal.pone.0184500

**Published:** 2017-09-27

**Authors:** Julie M. Lander, Dorothy M. Supp, Hua He, Lisa J. Martin, Xiaoting Chen, Matthew T. Weirauch, Steven T. Boyce, Raphael Kopan

**Affiliations:** 1 Division of Developmental Biology, Department of Pediatrics, Cincinnati Children's Hospital Medical Center, Cincinnati, Ohio, United States of America; 2 Research Department, Shriners Hospitals for Children, Cincinnati, Ohio, United States of America; 3 Department of Surgery, University of Cincinnati, Cincinnati, Ohio, United States of America; 4 Division of Human Genetics, Department of Pediatrics, Cincinnati Children's Hospital Medical Center, Cincinnati, Ohio, United States of America; 5 Center for Autoimmune Genomics and Etiology, Cincinnati Children’s Hospital Medical Center, Cincinnati, Ohio, United States of America; 6 Division of Biomedical Informatics, Department of Pediatrics, Cincinnati Children’s Hospital Medical Center, Cincinnati, Ohio, United States of America; University of Alabama at Birmingham, UNITED STATES

## Abstract

To identify putative gene regulatory regions that respond to epidermal injury, we mapped chromatin dynamics in a stratified human epidermis during barrier maturation and disruption. Engineered skin substitutes (ESS) cultured at the air-liquid interface were used as a model of developing human epidermis with incomplete barrier formation. The epidermal barrier stabilized following engraftment onto immunocompromised mice, and was compromised again upon injury. Modified formaldehyde-assisted isolation of regulatory elements (FAIRE) was used to identify accessible genomic regions characteristic of monolayer keratinocytes, ESS *in vitro*, grafted ESS, and tape-stripped ESS graft. We mapped differentiation- and maturation-associated changes in transcription factor binding sites enriched at each stage and observed overrepresentation of AP-1 gene family motifs in barrier-deficient samples. Transcription of *TSLP*, an important effector of immunological memory in response to allergen exposure, was dramatically elevated in our barrier-deficient samples. We identified dynamic DNA elements that correlated with *TSLP* induction and may contain enhancers that regulate *TSLP*. Two dynamic regions were located near the *TSLP* promoter and overlapped with allergy-associated SNPs rs17551370 and rs2289877, strongly implicating these loci in the regulation of *TSLP* expression in allergic disease. Additional dynamic chromatin regions ~250kb upstream of the *TSLP* promoter were found to be in high linkage disequilibrium with allergic disease SNPs. Taken together, these results define dynamic chromatin accessibility changes during epidermal development and dysfunction.

## Introduction

A major role of the epidermis is to act as a barrier that protects against external pathogens and prevents surface water loss. Disruption of the barrier induces an immune response to destroy invading organisms and create immunological memory against specific antigens. Dysregulation of this process can involve ectopic activation of the Th2 immune system and lead to allergic diseases including atopic dermatitis, asthma, and allergic rhinitis. Downstream of barrier disruption are a series of cellular responses, including changes in gene expression. Analysis of chromatin accessibility dynamics can identify potential regulatory regions that mediate the transcriptional response and provide insight into the mechanisms that link barrier dysfunction with immune sensitization.

Key components of the epidermal barrier include cross-linked lipids and proteins, such as Filaggrin, Loricrin, and late cornified envelope (LCE) proteins. When the physical barrier is breached, the epidermis also acts as a first-response immune organ. Keratinocytes below the barrier express pattern recognition receptors (e.g., Toll-like receptors); when stimulated by pathogens or allergens they produce of cytokines, including interleukins IL-1, -6, -25, and -33, and thymic stromal lymphopoietin (*TSLP*), to activate the local immune system [1–3]. Activated T-cells within the epidermis and dermis create a local immunological memory and produce additional cytokines that can further affect epidermal structure [4] and recruit circulating immune cells [5]. Ectopic immune activity can occur in the context of a barrier defect and lead to pathologic sensitization.

Loss of epidermal barrier integrity and over-activation of the Th2 arm of the immune system are characteristic features of atopic dermatitis (AD). In human disease, the progression from atopic dermatitis to asthma to allergic rhinitis is often referred to as the “atopic march” [6] and occurs in approximately 35–50% of children with AD [7]. TSLP is a reliable marker of barrier disruption [8] and has been strongly implicated in the pathogenesis of AD, allergic sensitization, and asthma [9, 10]. There are two protein-coding isoforms of *TSLP* in humans: the short form (_*sf*_*TSLP*) is constitutively expressed in skin and may act as an anti-microbial protein [11]. The long form of *TSLP* (_*lf*_*TSLP*) is an IL7–like cytokine that binds to a receptor dimer consisting of the IL7 receptor alpha chain and TSLP receptor [12]. In humans, _lf_TSLP acts directly on dendritic cells, type 2 innate lymphoid cells, and eosinophils, and promotes conversion of naïve T-cells into Th2 CD4 cells to create systemic immunological memory for an antigen [13]. Blocking _lf_TSLP signaling in mice with epidermal barrier defects prevents the progression from skin disease to airway inflammation and hyperreactivity [14–16]. While many mechanisms regulating _*lf*_*TSLP* transcription have been proposed [6, 17], it remains unclear which of these are most relevant to the development of atopic dermatitis.

To investigate the molecular pathways operating during differentiation and directly upstream of epidermal injury responses, including _*lf*_*TSLP* induction (henceforth referred to as *TSLP*), we analyzed genome-wide chromatin accessibility during multiple stages of epidermal barrier development and disruption. Changes in chromatin accessibility reflect an accumulated effect of covalent histone modifications [18], nucleosome repositioning [19], chromatin looping [20], and transcription factor binding, with open regions more likely to be active [21]. This information can be used to identify genomic loci that regulate gene expression [18, 19, 22–24]. To identify these regions, we modified the formaldehyde-assisted isolation of regulatory elements (FAIRE) protocol [25] to successfully recover and sequence open DNA fragments from stratified squamous human epidermis and assess changes in the genomic landscape associated with epidermal barrier development and dysfunction.

## Results

### An engineered skin substitute model for barrier disruption in human skin

**Fig 1:**
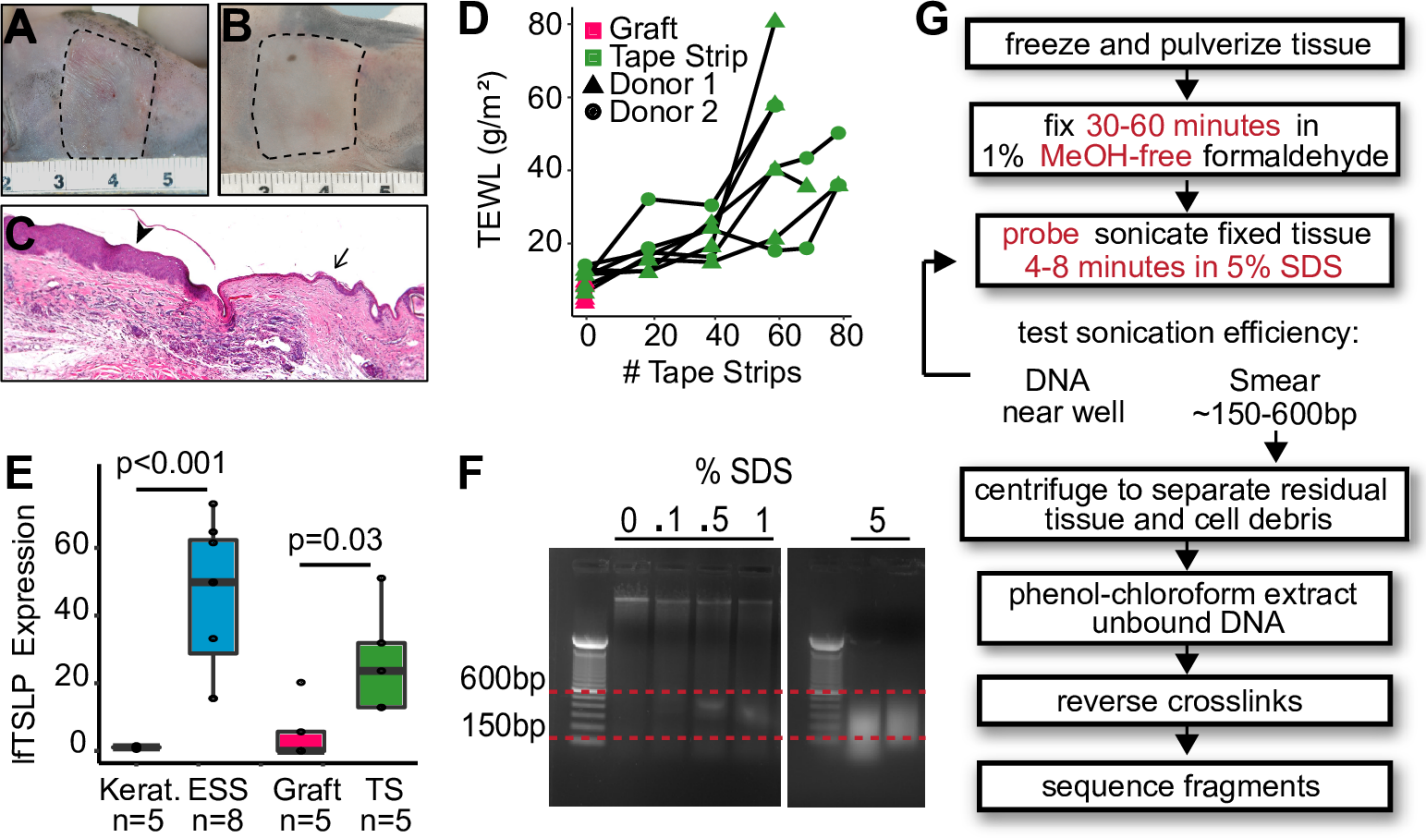
Engineered skin substitute (ESS) as a model to study human barrier maturation and injury. (A) ESS derived from primary human keratinocytes and fibroblasts were grafted onto 14 female NIH-III mice after two weeks in air-liquid interface (ALI) culture. Image was taken two weeks after surgery when bandages were removed. (B) Grafts were allowed to heal for an additional four weeks. (C) Histology of grafted ESS at the junction of the grafted mouse epidermis. Arrowhead indicates grafted human ESS, arrow points to native mouse skin. (D) Tape-stripping was performed on 7 of the mice, with the other 7 as grafted controls. Transepidermal water loss (TEWL) was used as an indicator of barrier integrity. (E) qRT-PCR using long-form *TSLP* -specific primers of monolayer keratinocytes, ESS at ALI, grafted ESS, and in grafted ESS 3 hours after tape-stripping. Data are normalized to GAPDH and shown as fold change over keratinocytes; error bars = standard deviation. (F) Sonicated ESS DNA run on a 1% agarose gel. Sonication was performed in buffer containing 0, 0.1%, 0.5%, 1%, and 5% SDS. (G) FAIRE workflow for human epidermis. Modifications from the published protocol are shown with red text.

In order to study chromatin dynamics in epithelial development, we asked whether engineered skin substitutes (ESS) could serve as a model of human skin during barrier maturation and disruption. ESS consist of stratified primary human epidermal keratinocytes cultured on dermal fibroblasts at the air-liquid interface (ALI) [26, 27] and can be stabilized by grafting onto immunocompromised mice (Fig 1A–1C, Methods). To induce an acute mechanical barrier defect, tape-stripping was performed. As the number of tape strips increased, we observed progressive disruption of the epidermal barrier as measured by TEWL (Fig 1D). ESS grafted on immunocompromised mice demonstrated low TEWL and barely detectable *TSLP* (Fig 1D and 1E). Barrier disruption by tape-stripping was associated with increased levels of *TSLP* three hours following injury (Fig 1E). Expression of _*sf*_*TSLP* was not significantly changed in our system (S1 Fig). Therefore, ESS grafts provide an adequate model of human epidermis and enable the study of *TSLP* regulation during ESS maturation and as triggered by human epidermal barrier disruption.

### Characterization of DNA elements identified by FAIRE

**Fig 2:**
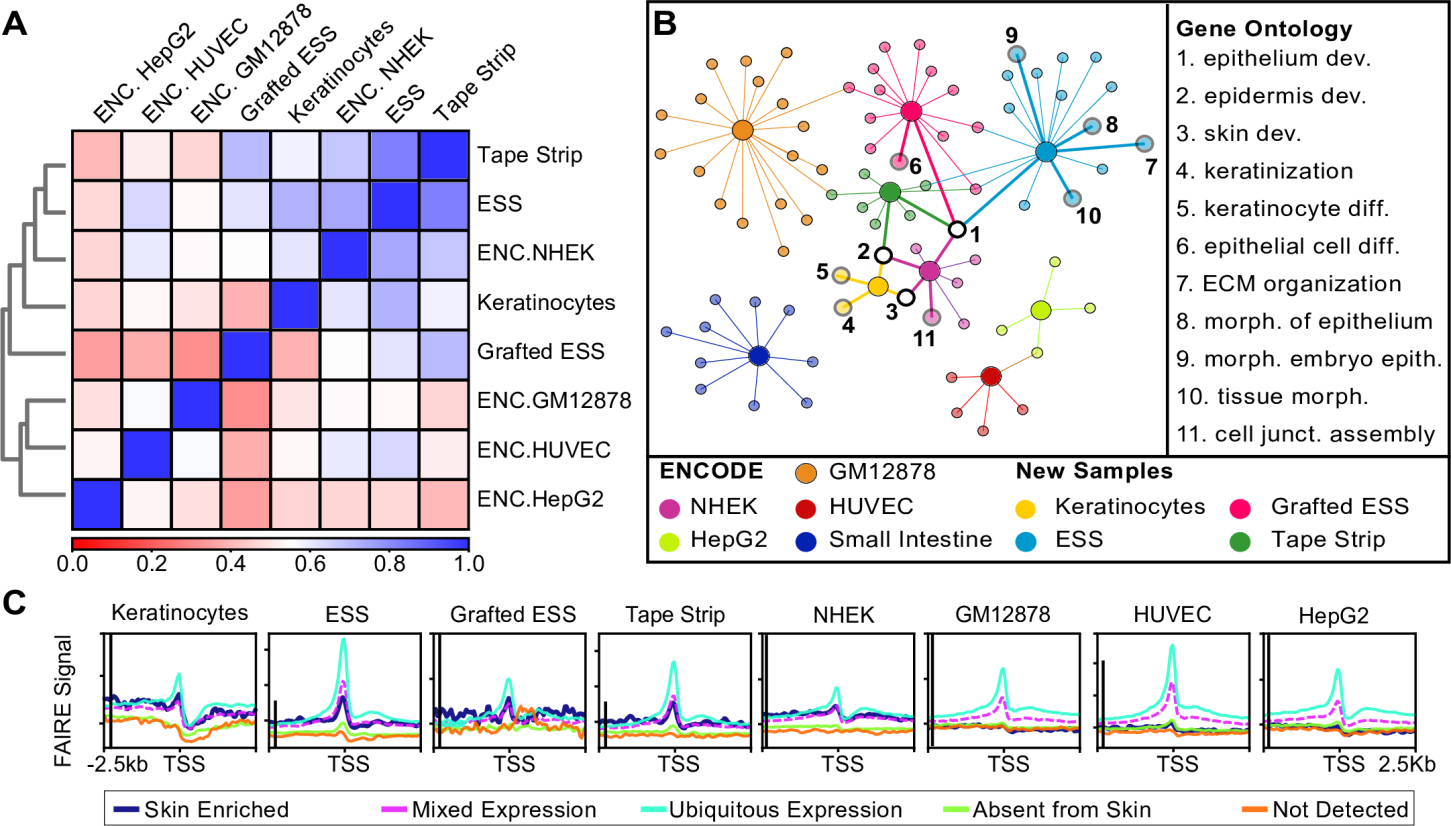
Global functional analysis of FAIRE signal and peaks. (A) Correlation matrix of FAIRE signal profiles in keratinocytes, ESS, Grafted ESS, Tape-stripped graft, and ENCODE NHEK, HUVEC, HepG2, and GM12878, using deepTools multiBigwigSummary. Blue represents perfect overlap, red indicates no correlation (see key). (B) ToppCluster analysis of the genes annotated to each FAIRE peak in Keratinocytes (yellow), ESS (light blue), Grafted ESS (pink), Tape-stripped ESS (dark green) and ENCODE NHEK (purple), HepG2 (light green), Small Intestine (dark blue), HUVEC (red), and GM12878 (orange) samples. Genes present in all samples were filtered out prior to analysis. Large circles represent an input sample and smaller circles represent GO terms. GO terms related to epidermal development are numbered and identified. (C) FAIRE signal within +/- 2.5kb of the transcription start site (TSS) of Human Protein Atlas lists of genes elevated in skin (dark blue), with mixed expression patterns that include skin (pink), expressed in all tissues (light blue), not expressed in skin (green), and not detected in any tissue type (orange). The y-axis scales on the ESS, Tape-strip, and HUVEC graphs were adjusted to better display the data. The black line to the right of the axis represents 3 arbitrary units of data coverage in each graph. Abbreviations: ENC- ENCODE; dev.- development; diff.- differentiation; ECM- extracellular matrix; morph.- morphogenesis; junct.-junction; epith.- epithelium.

Protease activity is known to induce *TSLP* expression [28–30]. Accordingly, we avoided methods such as ATAC-seq, which require isolation of single cells using Dispase or Trypsin that could result in upregulation of *TSLP* mRNA. To capture the chromatin conformation in ESS without introducing excessive experimental artifacts, we chose to use formaldehyde-assisted isolation of regulatory elements (FAIRE) [24, 31, 32], with longer fixation times and harsher sonication conditions as described in Methods and Fig 1G. To determine whether the FAIRE-seq DNA fragments reflected tissue-specific genomic features, we compared the FAIRE signal profiles obtained from Keratinocytes, ESS, Grafted ESS, and Tape-stripped graft, as well as ENCODE NHEK (normal human epidermal keratinocytes). As negative controls we included ENCODE FAIRE data for HepG2 (hepatocytes), HUVEC (human umbilical vein endothelial cells), and GM12878 (B-cells; [33]). Using deepTools multiBigwigSummary [34] we generated a correlation matrix of FAIRE results that reflected known cell- and tissue-type relationships: as expected, NHEK, Keratinocyte, ESS, Grafted ESS, and Tape-stripped samples were the most similar to each other, with HUVECs, HepG2, and GM12878 B-cells showing the least correlation with ESS samples (Fig 2A).

To determine whether FAIRE peaks were associated with tissue-specific gene expression, we obtained lists of genes from the Human Protein Atlas [35] that either (1) were elevated in skin, (2) showed mixed expression patterns in multiple tissues including skin, (3) were expressed in all tissues, (4) were not expressed in skin, or (5) were not detected in any tissues in the atlas. Analysis of FAIRE sequencing coverage within +/- 2.5kb from the transcription start site (TSS) for each of these genes demonstrated increased signal density near genes expressed in all tissues, genes with mixed expression, and genes elevated in skin (Fig 2C). Similarly, ENCODE FAIRE signals from GM12878, HUVECs, and HepG2 cells were not enriched around skin-specific genes. As a control, we performed the same analysis using genes expressed in spleen or brain. FAIRE signal for all cell types was absent from the vicinity of those tissue-specific genes, with the exception of GM12878 cells, whose signal was enriched near genes elevated in spleen (S2 Fig).

We next asked whether the genes associated with FAIRE peaks also reflected tissue-specific enrichment. Homer annotation [36] was used to assign a gene to each FAIRE peak. Gene ontology (GO) term analysis of these nearest neighbors revealed enrichment for several epidermal GO categories, including ‘skin development’, ‘epidermis development’, and ‘keratinocyte differentiation’. These terms were not enriched in the nearest neighbors of FAIRE peaks from ENCODE HepG2, small intestine, HUVECs and GM12878 cells (Fig 2B).

### Modified FAIRE identifies chromatin dynamics in human epidermal barrier maturation and function

**Fig 3:**
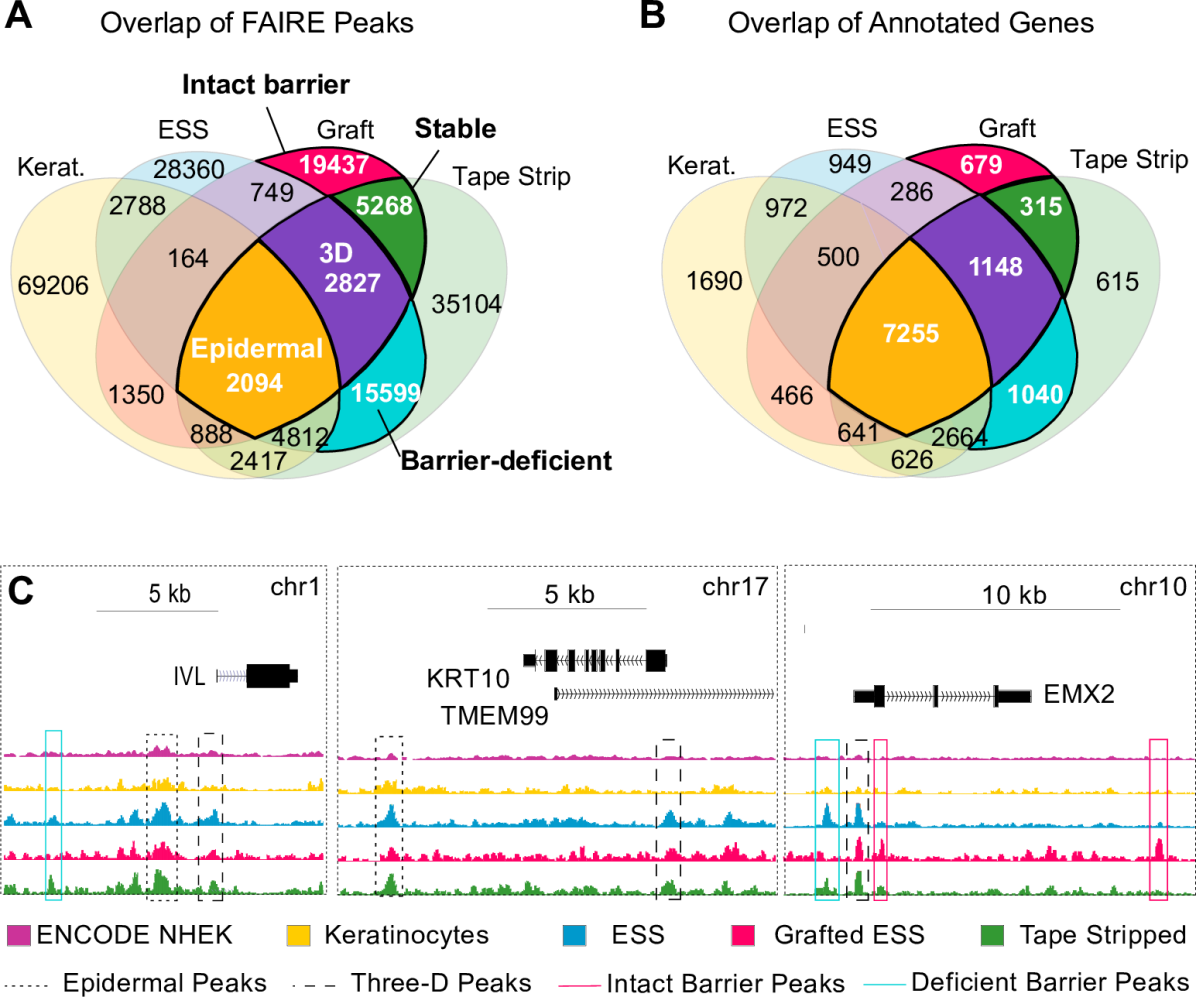
Dynamic FAIRE peaks in barrier maturation and function. (A) Venn diagram demonstrating overlap of FAIRE peaks in each sample and 5 relevant peak classes based on tissue pattern: Epidermal (n = 2094), Three-dimensional (3D; n = 2827), Stable (n = 5268), Intact Barrier (n = 19437), and Barrier-deficient (n = 15599). Peaks present in any non-epidermal cell type were filtered out prior to analysis. (B) Venn diagram showing overlap of annotated genes for each sample type. The nearest gene was assigned to each peak using Homer annotatePeaks. (C) FAIRE signals with representative peaks from four categories: Epidermal, dotted line; Three-dimensional, dashed line; Intact barrier, pink line; and Barrier-deficient peaks, blue line.

The analyses described above indicated that the FAIRE signals we obtained reflected human epidermal chromatin accessibility. To more specifically analyze chromatin structure at each stage, we filtered out FAIRE peaks shared with the ENCODE non-keratinocyte cell types listed in S3 Table. The remaining regions were categorized as: Epidermal (found in cultured Keratinocytes as well as differentiated ESS); Three-dimensional (3D; identified in ESS and Graft but not Keratinocytes); Stable (present in Graft and Tape-stripped graft, but not *in vitro* ESS); Intact barrier (found in Graft but not ESS *in vitro* or Tape-stripped graft); and Barrier-deficient epidermis (identified in Tape-stripped graft and *in vitro* ESS, but not in uninjured graft) (Fig 3A). Most FAIRE peaks were specific to their respective tissue type, which prompted us to analyze the overlap of the annotated genes for each peak. As shown in Fig 3B, the majority of genes were shared among samples. Representative peaks from each class are shown in Fig 3C. GO analysis of these regions using the GREAT analysis tool [37] revealed distinct GO enrichment for “molecular function” and/or “biological process”, as shown in S2 Table. Epidermal and 3D peaks were distinctly enriched near genes associated with epidermal growth and differentiation. Once we filtered these peaks from the Stable category, the GO terms were highly enriched for DNA-binding factors that modulate transcription. Notably, there was little enrichment for skin-related processes, but rather for melanocyte, skeletal muscle, and neural development. Similar terms were identified in the intact barrier sample. Finally, GO analysis of genes near peaks in Barrier-deficient samples revealed high enrichment for cytoskeletal rearrangement, including Rho-GTPase activation, actin filament assembly, collagen catabolism, and extracellular matrix degradation (S2 Table). These results coincide well with a wound response expected in damaged skin three hours following barrier injury.

### Differential enrichment of transcription factor binding sites

**Fig 4:**
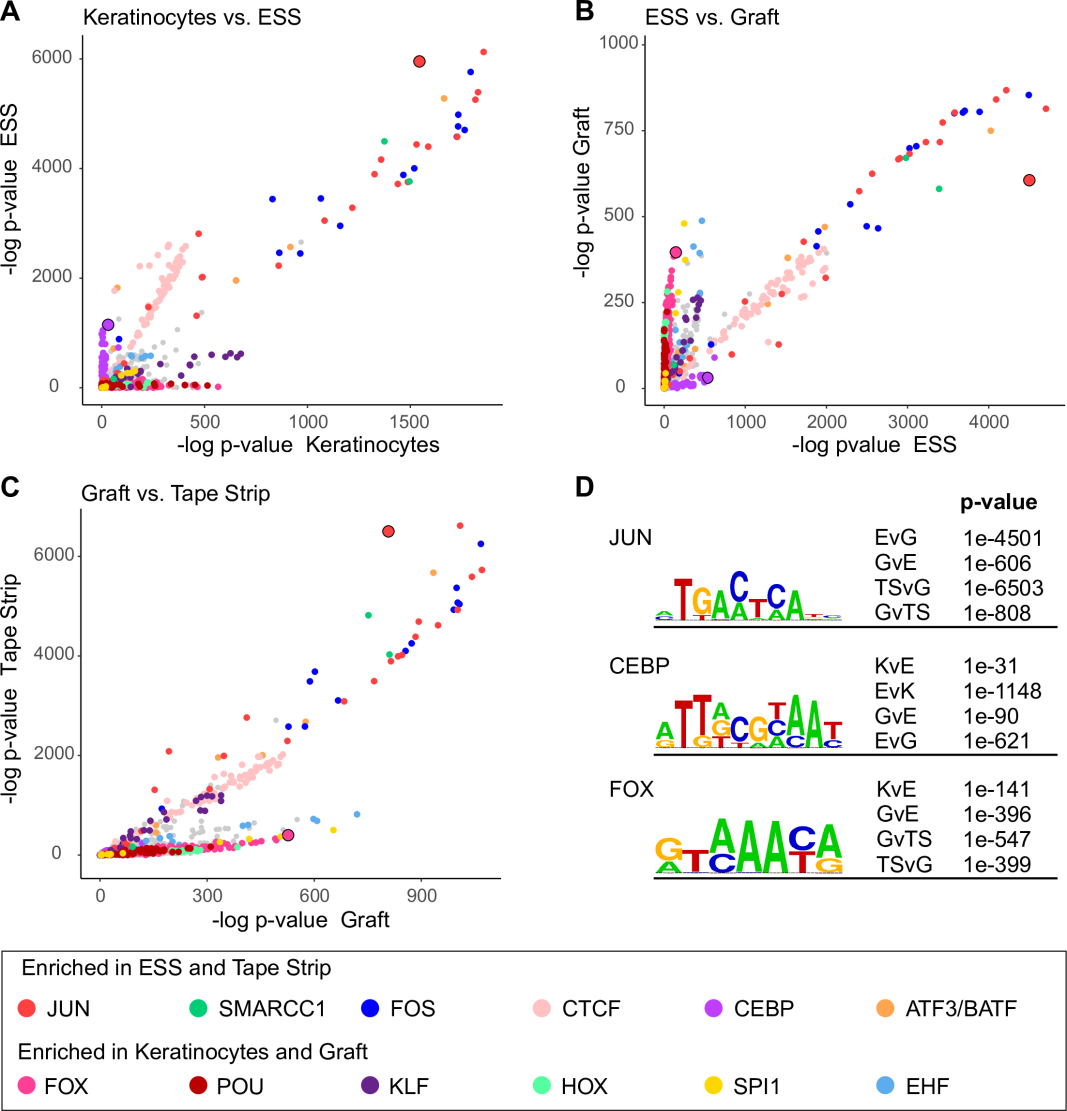
Transcription factor (TF) motif enrichment in chromatin associated with different stages in epidermal development or function. (A) Negative log of the p-value of over 3000 TF binding motifs from the Cis-BP catalog in Keratinocytes (x-axis) and ESS (y-axis). P-value indicates motif enrichment over a scrambled dinucleotide background (see text for details). Gene families with high significance are color-coded as shown in the legend. (B) Comparison between ESS and Grafted ESS and (C) Grafted ESS and Tape-stripped graft. (D) Representative motifs identified in a direct comparison of enrichment between data sets: ESS and Keratinocytes (EvK), ESS and Graft (EvG), Tape-strip and Graft (TSvG), Keratinocytes and ESS (KvE), Graft and ESS (GvE), and Graft and Tape-strip (GvTS). Full lists of motifs and p-values can be found in S1 Appendix.

These dynamic peaks represent potential regulatory regions that respond to barrier damage and induce transcriptional responses. To investigate which molecular pathways might be involved in this process, we analyzed transcription factor (TF) binding motifs specifically enriched under dynamic peaks in basal Keratinocytes, ESS, Grafted ESS, or Tape-stripped graft using Homer’s motif enrichment software [36] and the CIS-BP motif database [38]. The negative log of the p-value for each motif was then plotted to compare two particular dynamic peak sets (Fig 4). These pairwise comparisons were performed for basal Keratinocyte vs. ESS (Fig 4A), ESS vs. Grafted ESS (Fig 4B), and Grafted ESS vs. Tape-stripped graft (Fig 4C). These analyses revealed several TF families that were more likely to have binding sites in either Keratinocytes (2D, mostly basal cells) and Graft (basal cells and stable supra-basal cells) or ESS and Tape-strip (both 3D but with incomplete barrier). Among those enriched in ESS and Tape-strip were JUN and FOS, components of the AP-1 transcription complex. CTCF and CEBP also showed a preference for ESS FAIRE peaks. Motifs more prevalent in Keratinocyte and Graft samples included binding sites for FOX, HOX, and POU protein families. Representative sequences are shown in Fig 4D, and complete results are available in S1 Appendix.

### Changes in chromatin dynamics within the *TSLP* locus and their association with allergy susceptibility loci

**Fig 5:**
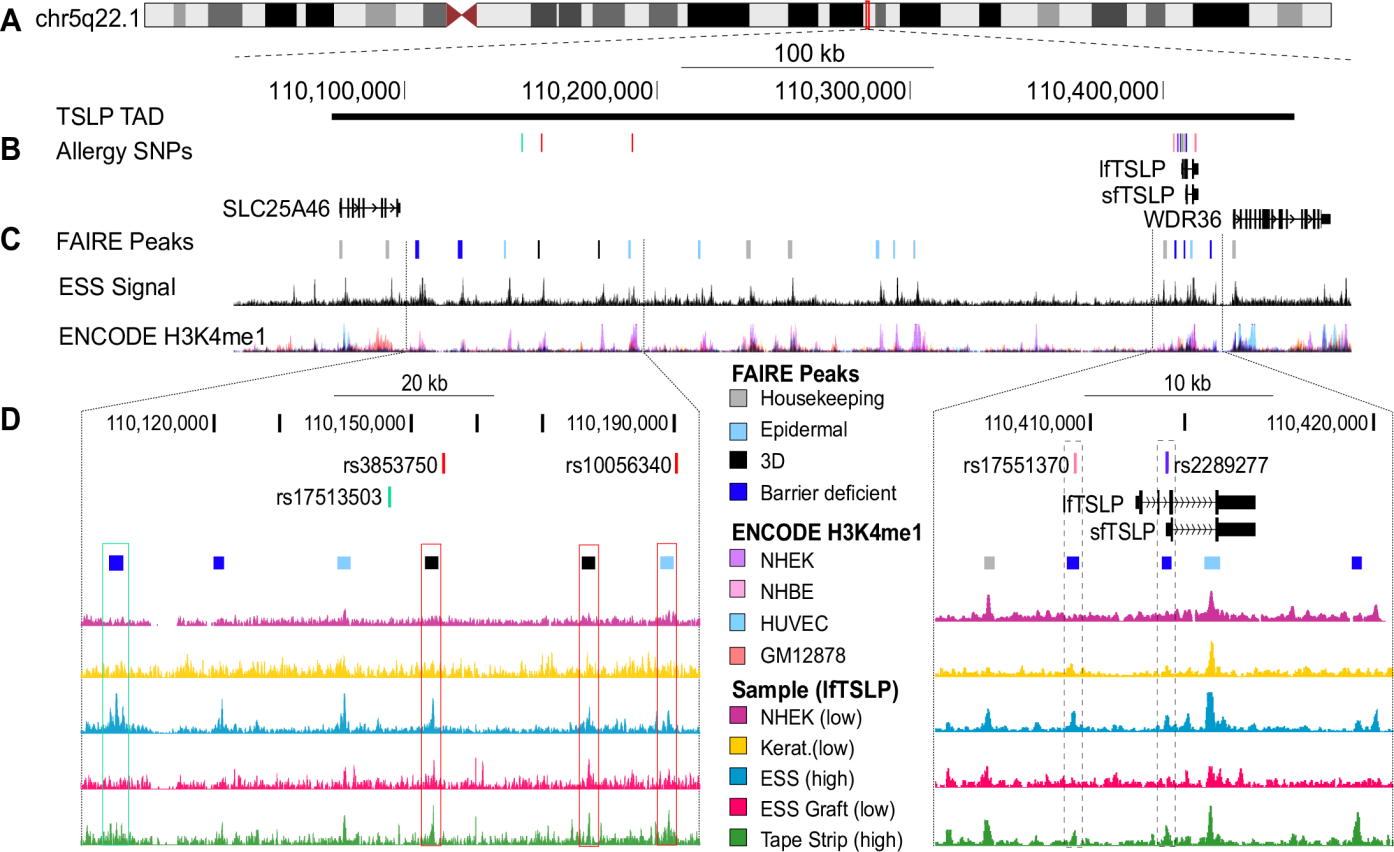
Dynamic FAIRE peaks in the *TSLP* transcriptionally associated domain (TAD) associate with allergic disease-associated SNPs. (A) The most likely *TSLP* regulatory region was identified using published Hi-C data from several cell types to infer the TAD around *TSLP* [39]. (B) GWAS SNPs located within the *TSLP* TAD (n = 16); all are associated with allergic disease (see Table 1 and Methods). (C) FAIRE peaks within the *TSLP* TAD designated by category: Housekeeping peaks which were present in our samples as well as in at least 3 ENCODE FAIRE cell types (grey); epidermal peaks, present in our samples but not in non-epidermal ENCODE cell types (light blue); Three-dimensional (3D) peaks present only in stratified epidermis (black); and Barrier-deficient peaks that were present in the context of insufficient epidermal barrier (ESS and Tape-strip) but absent in an intact barrier (Graft) or culture monolayer (keratinocytes, NHEK; dark blue). Representative ESS signal is shown below, along with ENCODE H3K4me1. Color designations for the most prominent cell types in the H3K4me1 track are indicated in the legend. (D) FAIRE signals from ENCODE NHEK, cultured Keratinocytes, ESS, Grafted ESS, and Tape-stripped ESS are shown for the *TSLP* locus (right panel) as well as a distal region ~200-250kb upstream of *TSLP* (left panel) containing allergy-associated SNPs and dynamic peaks. Two Barrier-deficient peaks (dashed boxed regions, right panel) overlap rs17551370 and rs2278277, respectively. Rs17551370 is associated with allergic rhinitis [42] and rs2289277 is a known expression quantitative trait locus (eQTL) [43] for *TSLP* and associated with changes in IgE levels [44, 45]. Four distal (left panel) Epidermal, 3D, and Barrier-deficient FAIRE peaks are in high (R^2^ = 0.8–1) linkage disequilibrium (LD) with allergy-associated SNPs. Peaks are boxed in the same color as the SNP they are in LD with. Full LD plots and analysis are in S4 Fig and Table 1.

The data above identified genomic chromatin regions responding to barrier injury. TSLP is a reliable early marker of barrier disruption [8]. To focus our search for putative, injury-responsive *TSLP* regulatory regions we inferred the most likely transcriptionally associated domain (TAD) for the *TSLP* locus on Ch5q22.1 using published Hi-C (genome-wide chromosome conformation) data obtained from multiple cell types [39] (Fig 5A). A TAD defines the regions of chromatin that are likely to contain the regulatory elements of the genes contained within it [40, 41]. The expression levels of other genes in this TAD (*SLC25A46*, *WDR36*, and _*sf*_*TSLP*) did not significantly change in our samples (S1 Fig), but the long isoform of *TSLP* was increased in both models of barrier deficiency (ESS and Tape-stripped graft; Fig 1E), suggesting that _*lf*_*TSLP* is the gene target of the putative dynamic enhancers we identified within this locus.

**Table 1:** Allergy-associated SNPS in LD with dynamic peaks in the *TSLP* TAD. Locations of dynamic peaks containing SNPs that are in LD with allergic disease associated SNPs. R^2^ indicates LD based on 1000 genomes phase 1 CEU data. Abbreviations: AR- allergic rhinitis; AD- atopic dermatitis; EoE- eosinophilic esophagitis; CR- chronic rhinitis; eQTL- expression quantitative trait locus.

Peak Location			Peak Class	SNP in Peak	Allergy SNP	R^2^	Association	Association Reference
chr5	110403882	110404338	Barrier-deficient	rs17551370	rs17551370	NA	AR, total serum IgE	[42]
					rs10062929	1	Asthma+AD, EoE, total serum IgE	[42, 45, 46]
					rs11466750	1	Asthma+AD	[46]
					rs11466749	0.85	AD, asthma, AR	[42, 46]
chr5	110408867	110409332	Barrier- deficient	rs2289277	rs2289277	NA	total serum IgE, eQTL	[43–45]
					rs3806932	0.95	EoE, CR, eQTL	[47]
					rs3806933	0.96	FA, asthma, eQTL	[47, 48]
chr5	110153049	110153553	3D	rs7735355	rs3853750	1	asthma, hay fever	[49]
				rs7735519	rs10056340	1	allergic sensitization	[50]
				rs7717615				
chr5	110104230	110106393	Barrier- deficient	rs17506794	rs17513503	0.87	allergic rhinitis	[51]
chr5	110176713	110177307	3D	rs72774897	rs17513503	0.88	allergic rhinitis	[51]
				r s1350294	rs3853750	0.82	asthma, hay fever	[49]
					rs10056340	0.82	allergic sensitization	[50]
chr5	110188917	110189371	Epidermal	rs7712806	rs17513503	0.88	allergic rhinitis	[51]

The FAIRE peaks located within the TAD fell into four general categories: Housekeeping peaks, which were present in our samples as well as in at least 3 ENCODE FAIRE data sets; Epidermal specific peaks, present in our samples but absent from non-epidermal ENCODE cell types; Three-dimensional (3D) peaks present only in stratified tissues; and Barrier-deficient peaks which were present in the samples with insufficient barrier function and *TSLP* expression (ESS and Tape-stripped graft) but were absent from stable epidermis with an intact barrier (Graft) or monolayer cell cultures that do not express *TSLP* (Keratinocytes, NHEK; Fig 5C). Epidermal peaks could represent enhancers that are necessary for _*sf*_*TSLP* and/or *TSLP* transcription. Notably, peaks of this class frequently overlapped with ENCOE NHEK-specific H3K4 monomethylation (Fig 5C).

As many genetic association studies select SNPs unrelated to functional potential, it is very possible that disease associated SNPs may simply segregate with functional variants. As such it is important to evaluate the degree of linkage disequilibrium (LD) between disease-associated SNPs and SNPs in this FAIRE peaks. To further delineate which peaks may be associated with allergic or atopic disease, we investigated the association between dynamic FAIRE peaks and specific single nucleotide polymorphisms (SNPs). Sixteen allergic phenotype- associated SNPs were identified within the *TSLP* TAD (Fig 5B, Table 1). The SNPs clustered in two regions: a proximal cluster near the *TSLP* locus and a distal cluster approximately 200-250kb upstream of *TSLP*. Two proximal SNPs (rs17551370 and rs2289277) were located within Barrier-deficient FAIRE peaks near the *TSLP* promoter (Fig 5D, right panel). When expanding the evaluation to SNPs in linkage disequilibrium (LD) with SNPs in the FAIRE peaks, we identified four additional disease-associated SNPs in the Barrier-deficient peaks: three in strong (R^2^ > 0.8) LD with rs17551370 and one in strong LD with rs2289277 (Table 1, S3 and S4 Figs). In the distal peak cluster, SNPs within 1 Barrier-deficient, 2 3D, and 1 Epidermal peak were in strong LD with disease-associated SNPs rs3853750, rs10056340, or rs17513503 (Fig 5D left panel, Table 1, S3 and S4 Figs). In total, 3 of 5 Barrier-deficient, 2 of 2 3D, and 1 of 9 Epidermal dynamic loci within the *TSLP* TAD were associated with at least one allergy-associated SNP. The dynamic nature and disease-association of these regions make them strong candidates for regulatory elements that influence TSLP induction in allergic diseases.

## Discussion

The skin is a complex organ that serves as the interface between an organism and its environment. Skin keratinocytes respond to epidermal disruption by producing a variety of pro-inflammatory cytokines, neurotransmitters, and hormones [52]. Defects in epidermal barrier development and maintenance are associated with chronic cytokine production, leading to cancer susceptibility in mice [53, 54] and a wide range of human diseases, including atopic dermatitis [55, 56]. Understanding changes in chromatin structure can reveal key regulatory elements that link the physical damage with a transcriptional response. Several studies have analyzed chromatin dynamics in keratinocytes during *in vitro* monolayer differentiation [57–59], but study of the epidermal barrier requires a stratified epithelium. As of this writing we are unaware of any genome-wide analysis of chromatin structure performed on an intact, stable human epidermis. Subsequently, the changes observed in our system are more likely to represent chromatin dynamics during *in vivo* human development and disease.

Our study revealed characteristic patterns of open chromatin during multiple stages of skin barrier development and function, starting with human keratinocytes grown in a monolayer, stratified at the air-liquid interface, and after stable engraftment *in vivo*. Interestingly, different stages of epidermal development were defined more by their chromatin signature than the genes annotated to each FAIRE peak. These results concur with those of Cavazza, et al. [59], who analyzed chromatin dynamics during *in vitro* keratinocyte differentiation and found that while there was considerable overlap in the promoters transcribed in progenitor and differentiating keratinocytes, the active enhancers were often unique to a particular stage. However, we did observe that when FAIRE peaks present in Keratinocytes and ESS were filtered from Stable graft data, the GO term enrichment for epidermis-related categories diminished, suggesting that chromatin near genes commonly associated with epidermal differentiation is already open in cultured keratinocytes. This interpretation is supported by the presence of histone 3 lysine 4 monomethylation (H3K4me1) in ENCODE NHEK [33], a histone signature often associated with poised regulatory regions.

Our injury model also induced unique changes in chromatin accessibility. Three hours after tape-stripping, FAIRE peaks appeared near genes involved in cell migration (regulators of Rho, cell-substrate adhesion, and Actin bundles disassembly) and ECM remodeling (collagen catabolic processes, ECM disassembly; S2 Table). These changes are consistent with a coordinate wound healing response coincident with upregulation of innate immune response genes such as antimicrobial peptides and a wide range of cytokines, including TSLP [60]. By contrast, comparing the graft *in vivo* to ESS *in vitro* revealed changes in maturation of melanocyte and patterns associated with neural development, which may be due to rare melanocytes, cells of neural crest origin that were undetectable in ESS *in vitro* but expanded after grafting to form spots of pigment visible in some grafts *in vivo* (Fig 1A and 1B).

Analysis of TF binding motifs enriched under FAIRE peaks revealed distinct patterns that differentiated Barrier-deficient samples from Keratinocytes and Stable graft. Cultured keratinocytes and Stabilized graft showed enrichment for several homeobox binding motifs, including HOX, FOX, and POU families. Closer investigation, however, reveals that while these families’ motifs are similarly elevated, several of the specific motifs enriched in Keratinocytes and Graft differ. This is not surprising, given that cultured keratinocytes represent undifferentiated basal cells, while the grafted ESS are the most stable of all our samples. In contrast, AP-1 motifs were particularly enriched in ESS and Tape-stripped graft. AP-1 is an important factor in multiple stages of epidermal development [61, 62], including direct regulation of genes in the epidermal differentiation complex [58]. AP-1 has also been shown to play an important role in epidermal response to injury, including re-epithelialization [63] and inflammation [64]. Functional AP-1 sites have been identified near the *TSLP* locus [65] and are implicated in long form *TSLP* induction [44].

TSLP is a key component in the inflammatory response to barrier damage. TSLP can induce immunological memory leading to the atopic march, and has proven potential in inducing anti-tumor immunity in mice [66, 67] and man [68]. However, it is unclear which mechanisms are responsible for translating barrier dysfunction to increased *TSLP* transcription in human atopic dermatitis [69]. *TSLP* can be induced by proteases [29, 46], Vitamin D receptor agonists [70–72], Toll-like receptor and NFkB activation [73–78], and NFAT signaling [79, 80]. However, previous data from our lab showed that in mice with chronic epidermal barrier defects, pathogens (activators of Toll receptors) [81] as well as IKK2 [82] are dispensable for TSLP production. Likewise, by using ESS we demonstrated that human *TSLP* could be induced in a sterile system containing only keratinocytes and fibroblasts (ESS). Performing grafting experiments on immunocompromised mice housed in a barrier facility neutralized the confounding influence of many immune cell-derived cytokines [3, 83], allergens, and pathogens. All of this suggests that while there are many paths to *TSLP* induction, it is unclear which are required for the increase in *TSLP* expression seen in human disease.

In our barrier-injury model, FAIRE identified several dynamic chromatin peaks within the *TSLP* TAD; Epidermal and 3D peaks could represent enhancers that are necessary for _*sf*_*TSLP* and/or *TSLP* transcription, and Barrier-deficient peaks may be responding to the sensor that detects epidermal barrier breach and induces *TSLP*. Two Barrier-deficient peaks were identified near the *TSLP* promoter, both of which overlapped allergy associated SNPs (rs17551370 [42] and rs2289277 [44, 45]); the latter is also an established expression quantitative trait locus (eQTL) of *TSLP* [43]. In both cases, the minor allele of these SNPs is protective against disease and, in the case of rs2289277, associated with decreased *TSLP* transcription. In addition, SNPs in the dynamic FAIRE peaks over 200kb upstream of *TSLP* were also in LD with allergy-associated SNPs suggesting that they may also contain *TSLP* regulatory elements. Further analyses into the regulatory mechanisms of *TSLP* in the context of a human barrier defect and atopic disease will refine these findings and establish whether all or only some of these regions are needed to sense barrier dysfunction, and which factors are needed to activate *TSLP* expression via these regions.

We successfully isolated open chromatin from a stratified, stable human epidermis and identified DNA regions uniquely associated with different stages in epidermal development and injury response. In particular, we identified key regions near the *TSLP* locus that likely play a role in regulating *TSLP* transcription in allergic disease. By looking at the intersection of allergic disease associated SNPs and Barrier-deficient peaks in our ESS model, we markedly reduced the number of regions which could contribute to regulation of TSLP after barrier injury. In conjunction with published gene expression profiles and chromatin dynamics of keratinocytes responding to calcium in 2D culture, these data will enable the assembly of a more complete image of the regulatory events during human epidermal differentiation, barrier maturation, and response to injury.

## Methods

### ESS culture

De-identified skin from surgical discard from reduction mammoplasty was collected from elective surgeries at the University of Cincinnati (UC) Medical Center. Because the samples were de-identified and no Protected Health Information (PHI) was collected, the UC Institutional Review Board (IRB) determined that this activity does not constitute human subjects research and is exempt from requirements for informed consent according to 45CFR46.101(b)(4). Engineered human skin substitutes (ESS) were prepared as previously reported [85] from 2 separate de-identified female cell donors. Briefly, primary human dermal fibroblasts were seeded at a density of 0.5x10^^6^/ cm^2^ on a collagen matrix. 24 hours later primary human epidermal keratinocytes were added at 1x10^^6^/ cm^2^. The collagen and cells were cultured at the air-liquid interface for 14 days in University of Cincinnati Dermatology Medium-1 (UCDM-1) with daily media changes [86].

### ESS grafting and tape- strip experiments

ESS were grafted onto 14 6–8 week old female Charles River NIH-III nude mice (*NIH-Lyst*^*bg-J*^*Foxn1*^*nu*^*Btk*^*xid*^). Mice were housed four to a cage in pressurized Individually Ventilated Cages with forced ventilation and water directly to each cage in the University of Cincinnati laboratory animal facility. They were provided with unrestricted access to standard chow and water. Deep bedding and nesting material were included in each cage.

Each ESS was cut into 2x2 cm squares and the squares were grafted onto full thickness excisional flank wounds [85, 87]. Mice were anesthetized with Avertin (tribromoethanol 25 mg/ml, 0.25–0.3 mg/g body weight) during surgery. The efficacy of anesthesia was determined by a lack of response to a toe pinch. While the mice were anesthetized, their eyes were covered in an ophthalmic ointment to prevent corneal dryness and trauma. To prevent infection cephtazidime (antibiotic) was administered 120 mg/kg IP at surgery.

For 14 days after surgery, while the mice remain in dressings, they were caged in Static Micro Isolator Cages with a filter lid and water bottle. They were fed with soft food (regular chow in water presented in the cage bed). Part (about half) of each cage was maintained on a heating pad to warm it; as a safety measure, part of the cage was not on the heating pad. Post-operative analgesics were not provided as these drugs alter or inhibit the wound healing process and would reduce the value of those studies. Additionally, the surgical site is a full thickness excision of skin with sharply-defined edges. Thus, the excised area no longer has functioning nerves and there is no gradient of injury between the excised region and surrounding skin. If the grafted sites are touched, the mice show no signs of distress.

Post-operative mice were monitored daily by the investigator as well as an animal facility technician. Dressing materials were closely inspected for evidence of chewing or scratching, and mice were re-dressed if necessary. Any mice demonstrating signs of pain or distress including difficulty in breathing, reduced mobility, hunching, rapid weight loss, not eating, were euthanized and excluded from the study.

Bandages were removed 2 weeks post-surgery and the grafts were allowed to heal for an additional 4 weeks. 6 weeks after grafting, the mice were divided into two groups: untreated (stable graft, n = 7) and tape-stripped, n = 7. Grafts from each cell donor were divided evenly between the groups. For tape-stripping, mice were anesthetized with isoflurane, using the same toe-pinch standard as above along with ophthalmic ointment. Initial trans-epidermal water loss (TEWL) measurements were recorded with a VapoMeter (Delfin technologies; Kuopio, Finland). Fresh 0.75 in square strips of 3M Scotch Magic tape were applied and rapidly removed in succession, with breaks for TEWL measurements. Tape-stripping continued until TEWL readings either reached a plateau, exceeded 4 times the original TEWL measurement, or the number of tape strips equaled 80. None of the mice demonstrated any distress during or after the tape-stripping process. Tape-stripped mice were returned to sterile cages. Three hours later, mice were euthanized and 4 mm punch biopsies were collected and flash frozen in liquid nitrogen for future RNA isolation. Tissue at the intersection of human ESS graft and mouse skin was fixed in 4% PFA for histology analysis. The remaining graft tissue was removed and flash-frozen for FAIRE analysis. Animal studies were carried out in strict accordance with the recommendations in the Guide for the Care and Use of Laboratory Animals of the National Institutes of Health with the approval of the University of Cincinnati Institutional Animal Care and Use Committee.

### RNA isolation and qRT-PCR

To isolate RNA from ESS and skin samples, fresh or frozen tissue was dissociated with a pellet pestle in RLT lysis buffer (Qiagen ID: 79216) with 1% beta-mercaptoethanol and passed through a QIAshredder column (Qiagen ID: 79656). RNA was purified using the Qiagen RNeasy Mini kit (Qiagen ID: 74106). cDNA was synthesized using the SuperScript II reverse transcriptase (Invitrogen 18064–022) with 50–200 ng RNA. Quantitative PCR was performed with iTag Universal SYBR Green Supermix (BioRad 172–5121). Primer sequences are listed in S1 Table.

### FAIRE

Frozen ESS were pulverized to a fine powder in liquid nitrogen and thawed in 1 mL 1% methanol-free formaldehyde (16% formaldehyde diluted in PBS; ThermoScientific 28906). Samples were transferred to a 15 mL tube and an additional 7 mL 1% methanol- free formaldehyde was added. After 30-minute fixation at room temperature on a rotator, Glycine was added to a final concentration of 0.13 M and the sample placed on the room-temperature rotator for 10 minutes. The tubes were centrifuged for 3 minutes at 700 x g at 4°C and the pellet was resuspended and washed three times in cold PBS at 4°C for 5 minutes. After the final wash, the fixed tissue was resuspended in 1 mL of sonication buffer containing 5% SDS and 10 mM Tris-HCl pH 8.0; 1 mM EDTA; 0.5 mM EGTA, 100 mM NaCl; 0.33% TritonX-100; 0.4% Sarkosyl; 0.03% Deoxycholate. Samples were kept on ice and rotated through 4–8 sonication cycles using the Branson 250 Digital Sonifier with a 1/8 inch doublestep tip (Branson Ultronics 101063588 (sonicator), 101148063 (tip). Each cycle consisted of 1 minute of sonication split into 1 second on/ 1 second off intervals (2 minutes total per cycle) at an amplitude maximum of 50%. Following sonication a 50 μl aliquot was taken to test sonication efficiency. The aliquot was incubated at 55°C with ProteinaseK and RNase A (0.2 mg/ml each) for 1 hour to reverse the crosslinks. The DNA was isolated by phenol-chloroform extraction and ethanol precipitation and analyzed on a 1% agarose gel. Once DNA sonication reached the desired level of fragmentation (approximately 150–600 bp), the sonicated tissue was transferred to a 1.7 mL tube and spun for 10 minutes at 10,000 x g. The resulting supernatant containing cross-linked proteins and DNA was separated from the pellet containing leftover tissue and debris and transferred to a new vial. The FAIRE phenol-chloroform extraction and DNA isolation then followed as described in [31].

### DNA sequencing

FAIRE DNA sequencing libraries were assembled using the Illumina low throughput sequencing adaptors and indexing primers (see TruSeq DNA Sample Prep Guide Illumina 15026486 C). Following quantification with Q-bit (Qbit 2.0 fluorometer, Qbit dsDNA High Sensitivity Assay kit Invitrogen Q32854), 40 ng DNA were used for the sequencing library. Library preparation was as follows: DNA fragments underwent end repair with T4 DNA Polymerase (New England Biolabs (NEB) M0203L), Klenow DNA Polymerase (NEB M0210L), and T4 Polynucleotide Kinase (NEB M0201L). Following purification with Agencourt AmPure XP DNA-binding beads (Beckman Coulter A63880) in polyethylene glycol (PEG) at a 1.9:1 bead: DNA volume ratio, adenine bases were added to the ends of each fragment with Klenow exonuclease (NEB M0212L). Pre-annealed adaptors were ligated overnight at 16°C with T4 DNA Ligase (NEB M0202L). Unligated adaptors and adaptor dimers were removed with an AMPure XP bead purification (1.2:1 bead: DNA) performed twice. PCR following adaptor ligation was performed with Phusion High-Fidelity DNA Polymerase (ThermoScientific F-530L) using unique Illumina TruSeq DNA LT indexing primers for 12–14 amplification cycles. Following PCR, amplified DNA underwent a final AmPure XP bead purification at a 1.1:1 or 1:1 PEG to DNA volume ratio. A small amount was run on the Fragment Analyzer (Advanced Analytical) to confirm successful removal of adaptors and other small DNA fragments. Sequencing was performed on the Illumina Hi-Seq2500. The quality of the resulting FASTQ files was analyzed with FastQC (http://www.bioinformatics.bbsrc.ac.uk/projects/fastqc) and the reads were aligned to the human genome hg19 with Bowtie 2 using default parameters [88]. For the Graft and Tape-strip samples, we excluded DNA sequences contributed by surrounding mouse tissues by only analyzing reads that aligned to the human genome.

### Bioinformatic analysis

All sequence alignments and analyses were performed using the hg19 genome assembly. Four to six FAIRE.bam alignment files for each sample type (Keratinocytes, ESS, Graft, Tape-stripped graft) were merged to better differentiate consistent FAIRE peaks. Data were analyzed using Homer software [36] as well as the deepTools [34] and BEDTools [89] packages through the Galaxy interface (usegalaxy.org [90]). ENCODE datasets used are listed in S3 Table. For the correlation comparison, deepTools multiBigwigSummary calculated the average score for every region of the genome using a bin size of 10,000 bp and compared ENCODE samples with our Keratinocyte, ESS, Graft, and Tape-strip samples. For signal profile analysis, lists of genes elevated in skin, in skin with mixed expression, expressed in all tissues, absent from skin, and not detected in any tissue were obtained from the Human Protein Atlas [35]. To determine sample coverage near each gene subset, we used the deepTools compute matrix function with default analysis settings to compare each of the FAIRE bigwig files to the transcription start sites (TSS) of the UCSC canonical transcript of each gene [91]. These were then graphed using deepTools plot profile.

FAIRE peaks were identified using Homer findPeaks [36] with the following parameters: tbp (tags per base) = 1, peak size = 200bp, false discovery rate (FDR) = 0.05, local area filtering off (-L 0). Homer’s annotatePeaks feature was used to assign a gene to each FAIRE peak. For GO analysis in Fig 2, gene lists for each sample were then compared and the genes common among all samples were removed resulting in a series of “uncommon” genes for each sample type. These gene lists were entered into ToppCluster (toppcluster.cchmc.org, [92]), which calculated the similarity among samples and also with gene ontology (GO) lists in the category “biological process” with an FDR <0.05.

#### FAIRE peak subcategories and GO analysis

To define the 5 subclasses of FAIRE peaks (Epidermal, 3D, Stable, Intact barrier, Barrier-deficient), we first ran intersect from BEDTools [89] using the appropriate files to identify peaks with at least 25% overlap. Then we subtracted any regions that overlapped at least 10% with peaks that were to be excluded. A.bed file containing a concatenated list of ENCODE [33] FAIRE peaks from multiple cell types (listed in S3 Table) was subtracted to remove peaks not specific to keratinocytes. The resulting.bed files containing DNA regions associated with different stage(s) of epidermal development and function were then analyzed with the GREAT tool [37] using the default gene association rule: Basal+extension: 5000 bp upstream, 1000 bp downstream, 1000000 bp max extension, curated regulatory domains included. When analyzing GO results, we required an FDR < 0.05 and GO terms with at least 30 genes in their list.

#### Motif analysis

Motif analyses were performed using the Homer motif enrichment toolkit [37]. To generate an appropriate background set for comparison to the input set (while maintaining dinucleotide frequencies), we took the union of the two FAIRE peak sets under comparison, and scrambled the resulting set of sequences while maintaining dinucleotide frequencies (e.g., to compare Keratinocytes and ESS, each input set of peaks was compared to a background containing a scrambled set of all of the peaks in Keratinocytes and/or ESS). For our purposes, we modified the HOMER code to (1) use the CisBP motif library [38] containing ~3000 motifs instead of the default HOMER motif library containing 246 motifs; and (2) scan the entire peak region as opposed to only the center of each peak using the ‘-size’ option.

### Allergy-associated SNPs in relation to FAIRE peaks

Allergic disease-related SNPs were compiled using the NHGRI-EBI genome-wide association studies (GWAS) catalog [93] and from candidate gene studies [42–46, 48–51, 94–96]. Sixteen were found within the genomic coordinates of the *TSLP* TAD (Table 1). The locations of these SNPs were evaluated to determine if they localized within the identified *TSLP* FAIRE peaks. Variants which were localized within FAIRE peaks and were disease associated were considered to have strong evidence of disease relevant regulatory effect.

We estimated LD using R^2^ from 1000 Genomes phase 1 data of CEU (European) founders [97]. We defined strong linkage as R^2^ greater than 0.8. Variants which were in strong LD with disease associated variants and were within a FAIRE peak were considered to have putative evidence of disease relevant regulatory effect [98].

## Supporting information

S1 Fig qRT-PCR of genes within the *TSLP* TAD. *SLC25A46*, *WDR36*, as well as isoform specific primers for _*sf*_*TSLP*. T-test comparisons of expression levels of each transcript among samples revealed no statistically significant differences (p>0.05). PCR primer sequences are in S1 Table.
(TIF)

S2 Fig Epidermal FAIRE signal near spleen- or brain-specific genes. Analysis of FAIRE signal from Keratinocyte, ESS, Graft, and Tape-stripped ESS, as well as ENCODE NHEK, GM12878, HUVEC, and HepG2 cells, within 2.5 kb of the transcription start sites (TSS) of genes either: 1. elevated in a specific tissue, 2. with mixed expression in the designated tissue and others, 3. ubiquitously expressed, 4. not expressed in the specific tissue type, or 5. not detected in any tissue. Graphs for genes from spleen and brain are shown.
(TIF)

S3 Fig Linkage disequilibrium of allergy-associated SNPs and dynamic FAIRE peaks. The *TSLP* TAD showing SNPs that are associated with allergic disease (red) and SNPs located within in dynamic FAIRE peaks (black). Signal track from ESS is used to show peak location. Bold black SNPs are in LD (R^2^ > 0.8) with a disease-associated SNPs. Bold red SNPs are associated with disease and present in a dynamic peak. In the LD plot below, black represents an R^2^ of 1 (100%), and white is no linkage. Squares boxed in red indicate loci of high LD between a SNP in a dynamic peak and an allergy-associated SNP.
(TIF)

S4 Fig Linkage disequilibrium of allergy-associated SNPs and dynamic FAIRE peaks (focused). LD block as in S3 Fig showing only the subset of disease-associated SNPs (red) and SNPs in LD (black, bold). The number in the box represents R^2^ on a scale from 0–100, with black indicating a value of 100 (perfect linkage).
(TIF)

S1 Table qRT-PCR primer sequences. (TIF)

S2 Table Gene ontology (GO) of FAIRE peak subcategories. The GREAT tool [37] was used to analyze gene ontology of FAIRE peaks defined as “Epidermal”, “Three-dimensional”, “Stable”, “Intact barrier”, or “Barrier-deficient”. The 10 most significant results in the GO categories “Biological Process” and/or “Molecular Function” are shown for each along with the FDR Q-value.
(XLSX)

S3 Table ENCODE datasets used. (XLSX)

S1 Appendix Full motif analysis. (XLSX)

S2 Appendix Numerical data for Figs 1D, 1E, S1 and 2A. (XLSX)

## Note Added in Proof

As this manuscript was being reviewed, a genome-scale analysis of chromatin architecture during calcium-induced keratinocyte differentiation *in vitro [84]* reported that many promoter-enhancer contacts were pre-established in undifferentiated keratinocytes. This observation strengthens our conclusion that many differentiation-specific genes are embedded in chromatin already open within basal keratinocytes. In addition, capture Hi-C data recorded the presence of long-range interactions between some of the putative enhancers identified by FAIRE and the TSLP promoter. Enhancer/promoter interactions within stable, grafted human epidermis remain to be determined.
