# Supplementary figures and images for: Analysis of chromatin accessibility in human epidermis identifies putative barrier dysfunction-sensing enhancers
Source: PLoS One. 2017 Sep 27;12(9):e0184500. doi: 10.1371/journal.pone.0184500 (PMC5617145; doi:10.1371/journal.pone.0184500)

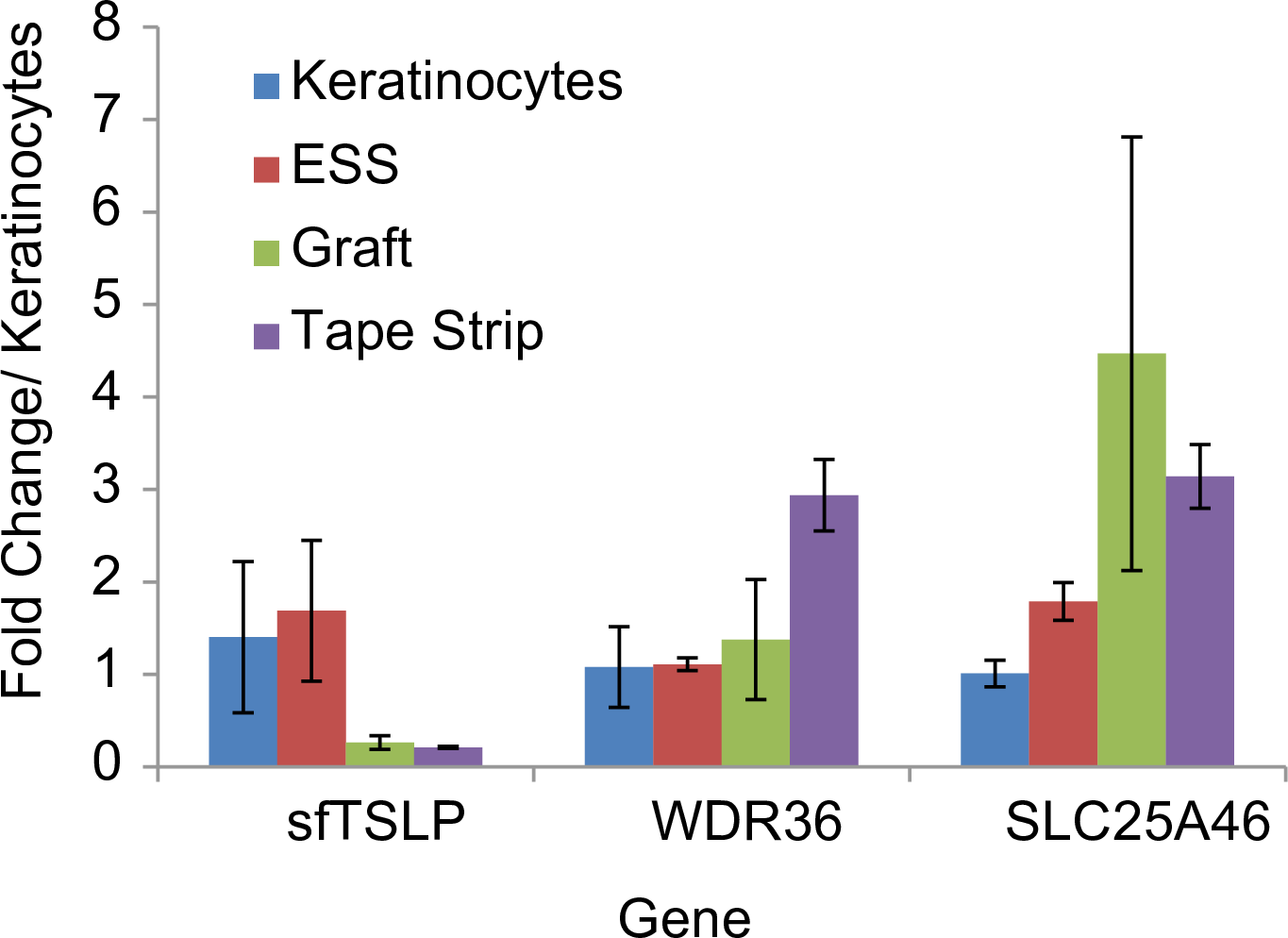

Supplement: S1 Fig — SLC25A46, WDR36, as well as isoform specific primers for sfTSLP. T-test comparisons of expression levels of each transcript among samples revealed no statistically significant differences (p>0.05). PCR primer sequences are in S1 Table. (TIF) [file pone.0184500.s001.tif]

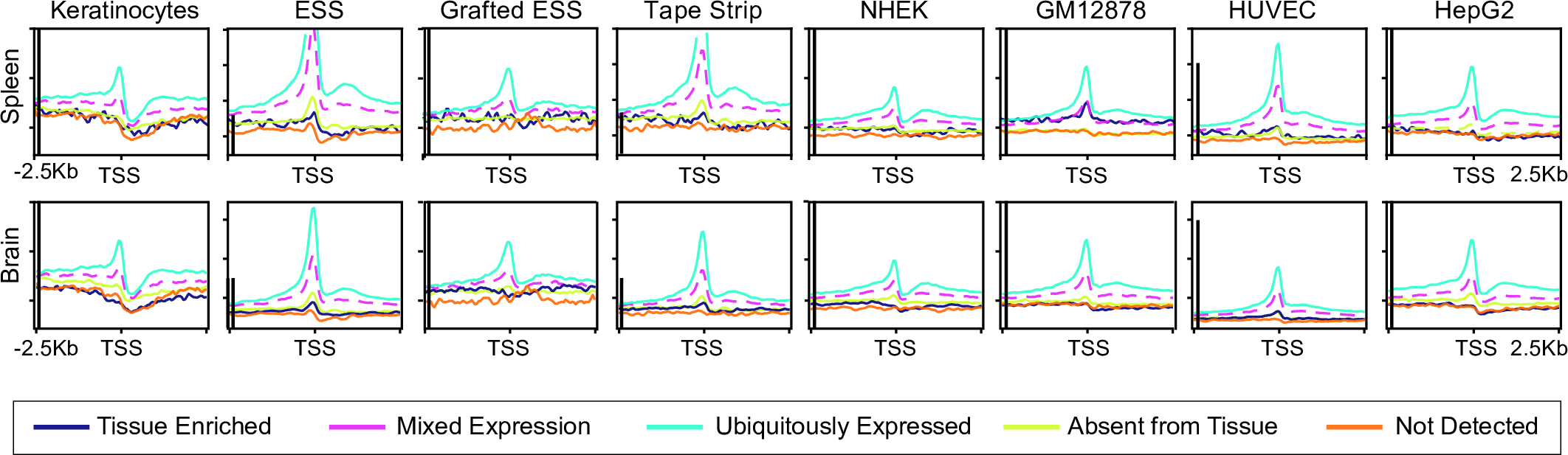

Supplement: S2 Fig — Analysis of FAIRE signal from Keratinocyte, ESS, Graft, and Tape-stripped ESS, as well as ENCODE NHEK, GM12878, HUVEC, and HepG2 cells, within 2.5 kb of the transcription start sites (TSS) of genes either: 1. elevated in a specific tissue, 2. with mixed expression in the designated tissue and others, 3. ubiquitously expressed, 4. not expressed in the specific tissue type, or 5. not detected in any tissue. Graphs for genes from spleen and brain are shown. (TIF) [file pone.0184500.s002.tif]

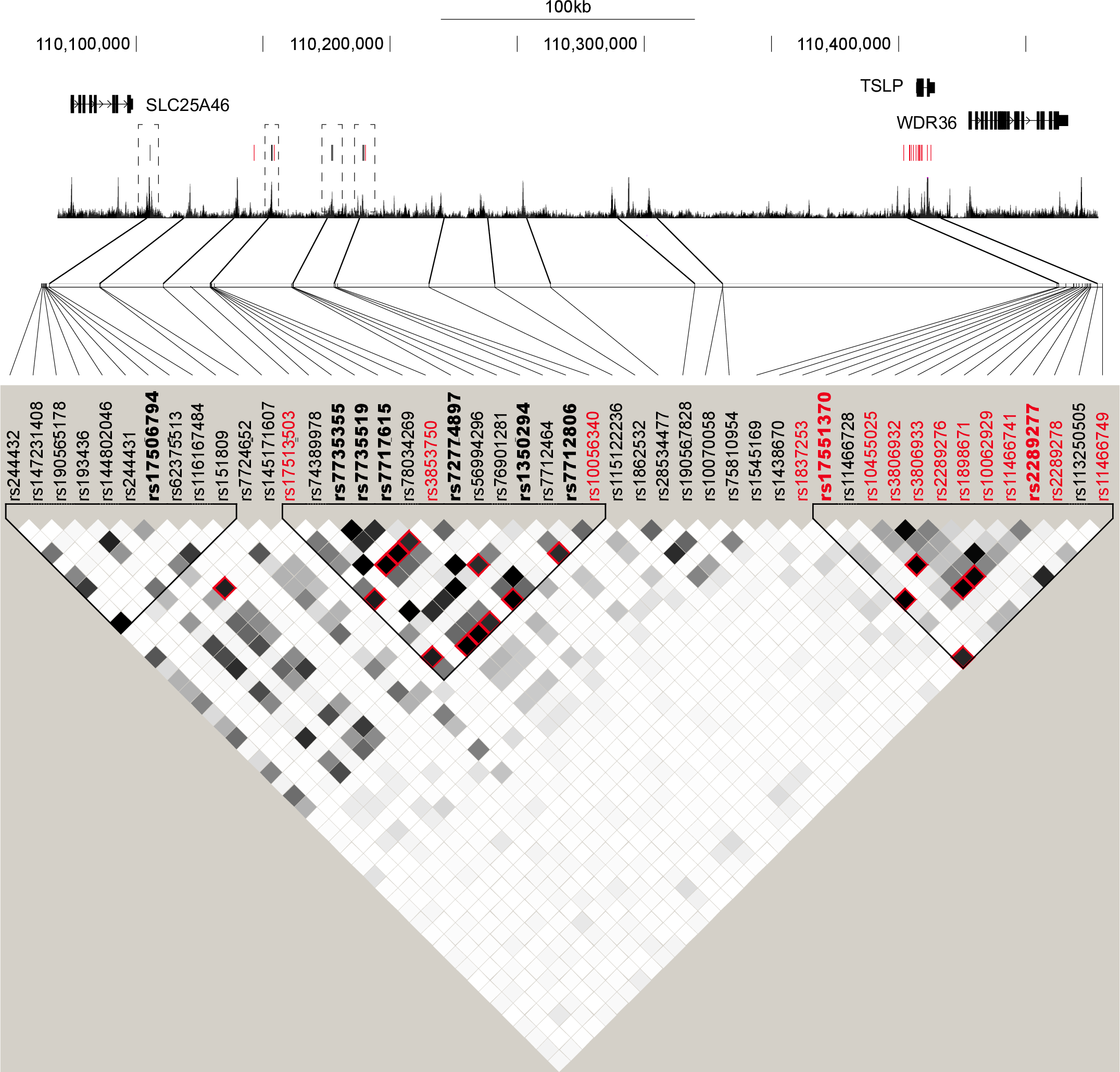

Supplement: S3 Fig — The TSLP TAD showing SNPs that are associated with allergic disease (red) and SNPs located within in dynamic FAIRE peaks (black). Signal track from ESS is used to show peak location. Bold black SNPs are in LD (R2 > 0.8) with a disease-associated SNPs. Bold red SNPs are associated with disease and present in a dynamic peak. In the LD plot below, black represents an R2 of 1 (100%), and white is no linkage. Squares boxed in red indicate loci of high LD between a SNP in a dynamic peak and an allergy-associated SNP. (TIF) [file pone.0184500.s003.tif]

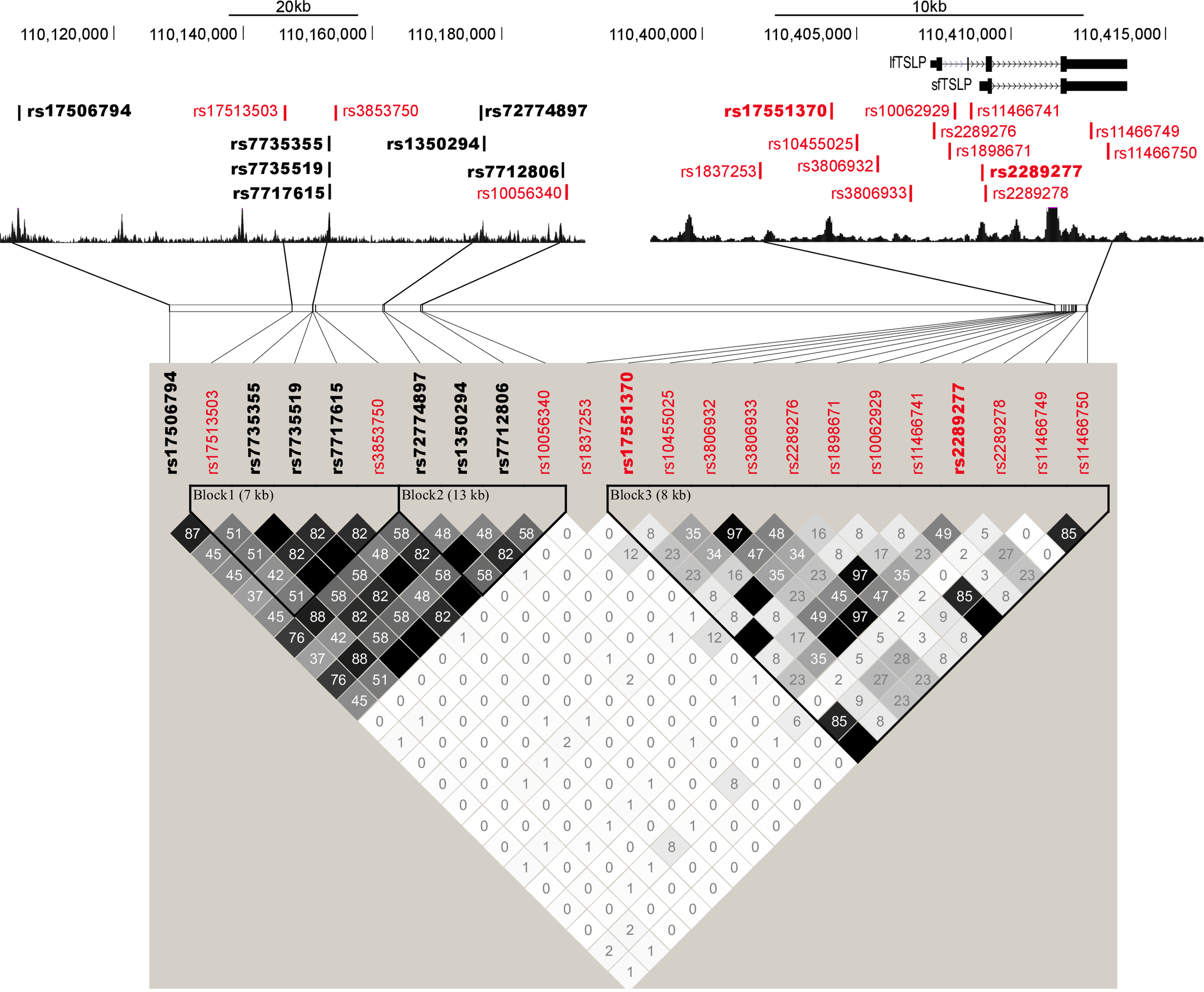

Supplement: S4 Fig — LD block as in S3 Fig showing only the subset of disease-associated SNPs (red) and SNPs in LD (black, bold). The number in the box represents R2 on a scale from 0–100, with black indicating a value of 100 (perfect linkage). (TIF) [file pone.0184500.s004.tif]

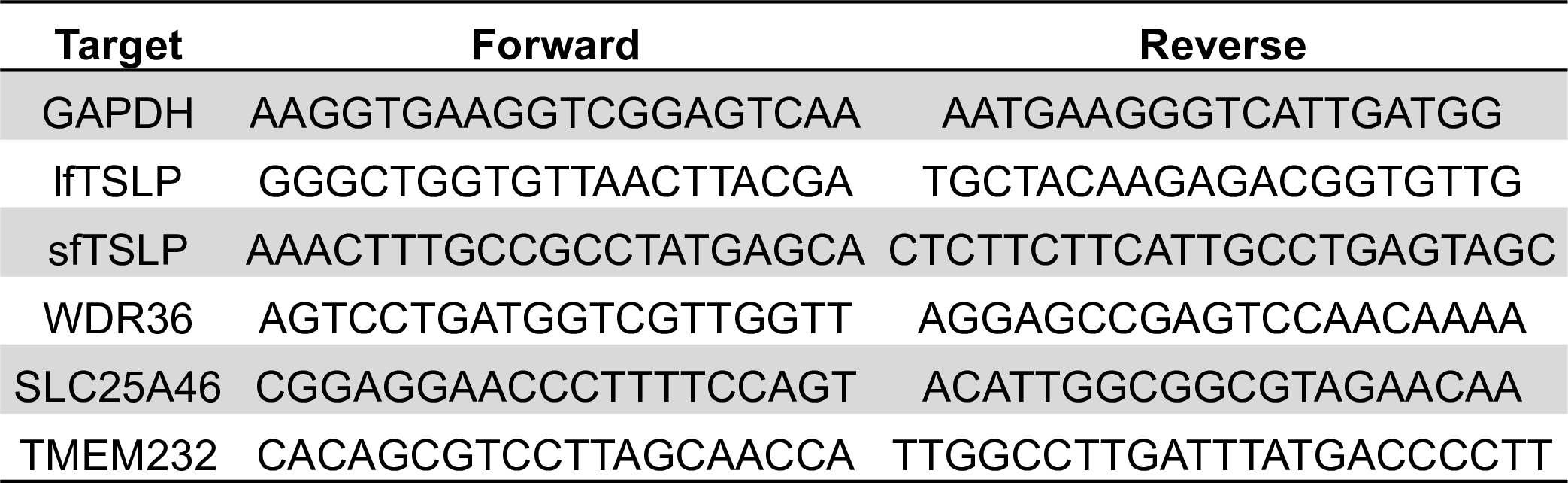

Supplement: S1 Table — (TIF) [file pone.0184500.s005.tif]
